# d-Amino Acids and Lactic Acid Bacteria

**DOI:** 10.3390/microorganisms7120690

**Published:** 2019-12-12

**Authors:** Jyumpei Kobayashi

**Affiliations:** Graduate School of Science, Technology and Innovation, Kobe University, 1-1 Rokkodaicho, Nada-ku, Kobe, Hyogo 657-8501, Japan; jyumpei.kobayashi@gmail.com

**Keywords:** d-amino acid, lactic acid bacteria, biofilm, enzyme, fermented food

## Abstract

Proteins are composed of l-amino acids except for glycine, which bears no asymmetric carbon atom. Accordingly, researchers have studied the function and metabolism of l-amino acids in living organisms but have paid less attention to the presence and roles of their d-enantiomers. However, with the recent developments in analytical techniques, the presence of various d-amino acids in the cells of various organisms and the importance of their roles have been revealed. For example, d-serine (d-Ser) and d-aspartate (d-Asp) act as neurotransmitters and hormone-like substances, respectively, in humans, whereas some kinds of d-amino acids act as a biofilm disassembly factor in bacteria. Interestingly, lactic acid bacteria produce various kinds of d-amino acids during fermentation, and many d-amino acids taste sweet, compared with the corresponding l-enantiomers. The influence of d-amino acids on human health and beauty has been reported in recent years. These facts suggest that the d-amino acids produced by lactic acid bacteria are important in terms of the taste and function of lactic-acid-fermented foods. Against this background, unique d-amino-acid-metabolizing enzymes have been searched for and observed in lactic acid bacteria. This review summarizes and introduces the importance of various d-amino acids in this regard.

## 1. d-Amino Acids

Proteins are basically made up of 20 kinds of α-amino acids in the form of monomers, and these amino acids have at least one asymmetric carbon, except for glycine. This yields 19 kinds of amino acid (glycine has been excepted) with l- and d-form varieties. As proteins are made up of l-amino acids, many researchers, in fields such as medical, food, and nutritional science, have paid close attention to l-amino acids, giving rise to considerable knowledge of l-amino acids, including proteins. Until the 1970s, many researchers thought that d-amino acids were rare in nature and did not play an important role in living organisms. After that, however, d-alanine (d-Ala), d-glutamate (d-Glu), and d-aspartate (d-Asp) were found in bacterial cell walls as constituents, and d-amino acids were observed to exist in nature. This finding led to the conclusion that d-amino acids were only rarely present in bacterial cell walls, but in the 1990s, the development of analytical techniques, such as chiral column chromatography, meant that various kinds of d-amino acids were identified in free forms in diverse organisms. These d-amino acids were initially considered to be derived from foods or enterobacteria, but it is now known that some parts of d-amino acids such as d-serine (d-Ser) and d-Asp can be synthesized in mammalian cells. d-Amino acids have similar physical and chemical characteristics with l-amino acids, but the physiological functions of d-amino acids are completely different with those of l-amino acids. For example, d-Ser works as neurotransmitter in the brain, but l-Ser has no such role. Furthermore, d-amino acids are uncommon in living organisms, compared with l-amino acids, and despite the fact that some d-amino acids have been found to play an important role in living organisms [1,2,3], aspects of their functions, uptake mechanisms, and metabolism are still unknown. Thus, the importance of d-amino acids is only now beginning to be recognized. Furthermore, recent studies have revealed that lactic acid bacteria produce a high quantity of various d-amino acids intra- and extracellularly and that these lactic acid bacteria harbor genes encoding unique d-amino-acid-metabolizing enzymes. This review, therefore, introduces the various functions of d-amino acids and their relationship with lactic acid bacteria.

## 2. Analytical Techniques of d-Amino Acids

As mentioned above, research related to d-amino acids has blossomed with the improvement of analytical techniques to study d-amino acids. To date, many studies employ high-performance liquid chromatography (HPLC) for determination of various kinds of d-amino acids. Kato et al. reported an HPLC method combining d-amino acid oxidase (DAO) and 1,2-diamino-4,5-methylenedioxybenzene (DMB) [4]. In this method, d-amino acids were oxidized to their corresponding 2-oxo acids by DAO, and these 2-oxo acids were nonenzymatically reacted with DMB. The resultant corresponding fluorescent compounds were determined by HPLC. This method achieved the determination of 18 kinds of d-amino acids during a 60 min run time. Furthermore, Gogami et al. succeeded in determining all d- and l-amino acids with an HPLC method using *o*-phthalaldehyde and *N*-acetyl-l-cysteine (OPA-NAC) as well as (+)-1-(9-fluorenyl) ethyl chloroformate and 1-aminoadamantane (FLEC-ADAM) derivatization methods at a maximum run time of 90 min [5]. Although these useful HPLC methods enable simultaneous analysis of various kinds of d-amino acids, relatively long run times were needed. Mutaguchi et al. employed ultraperformance liquid chromatography for determination of various d-amino acids and succeeded in determining 15 kinds of l-amino acids and 16 kinds of d-amino acids within 18 min of run time using OPA-NAC and OPA-*N*-*tert*-butyloxycarbonyl-l-cysteine derivatizations [6].

Enzymatic assays are another analytical method often suggested especially in d-Ser and d-Asp analysis because these d-amino acids play pivotal roles in mammalian cells. Ito et al. reported a rapid and simple d-Ser assay using serine dehydratase (SerDH) of *Saccharomyces cerevisiae* [7]. Although a lot of previously reported SerDHs degrade both d- and l-Ser into pyruvate, *S. cerevisiae* SerDH (ScSerDH) dominantly catalyzes d-Ser. In this method, pyruvate produced from d-Ser by a catalytic reaction of ScSerDH is spectrophotometrically assayed with lactate dehydrogenase and reduced nicotinamide adenine dinucleotide (NADH) or with 2,4-dinitrophenylhydrazine. Because of the unique characteristics of ScSerDH, a precise determination of d-Ser at the micromolar level comparable to the HPLC method was achieved. This enzymatic d-Ser assay method has since been improved into a more sensitive assay using pyruvate oxidase, horseradish peroxidase, and 10-acetyl-3,7-dihydroxyphenoxazine [8]. Furthermore, this method enables the assay of total Ser using mutant alanine racemase (AlaR) from *Geobacillus stearothermophilus*, which can racemize d- and l-Ser. Besides this enzymatic d-Ser assay, an enzymatic d- and l-Asp assay was also reported [9]. In this method, aspartate racemase (AspR), l-aspartate dehydrogenase (l-AspDH), and l-aspartate oxidase (l-AspO) were used. First, l-Asp in a sample was assayed by l-AspDH and NADH. Next, l-Asp was degraded by l-AspO and consequently d-Asp in the same sample was racemized to l-Asp. The l-Asp formed from d-Asp by AspR was assayed by l-AspDH and NADH, and the calculated l-Asp concentration was equivalent to the d-Asp concentration. As mentioned above, the importance of d-Ser and d-Asp for mammals has been recently revealed. Therefore, rapid and affordable assay methods for these d-amino acids using various enzymes have been suggested. However, the importance of other d-amino acids may be revealed in the future, and enzymatic assay methods for such d-amino acids may be developed.

## 3. d-Amino Acids in Mammals

d-Ser is an important and abundant substance that works as a neurotransmitter in the mammalian brain [10,11]. d-Ser functions as the coagonist of the *N*-methyl-d-aspartate receptor, which relates to the higher functions of the mammalian brain, and d-Ser has been reported to be involved in various diseases, such as schizophrenia [12,13] and amyotrophic lateral sclerosis [14]. Furthermore, it has been recently revealed that d-Ser is involved in synapse and dendrite formation in the brain [15,16]. d-Ser is generally synthesized in the brain from l-Ser via catalytic reaction of serine racemase (SerR), whereas SerR simultaneously possesses SerDH activity that degrades d- and l-Ser to pyruvate [17,18]. However, *SerR* knockout mice exhibited a decrease of d-Ser in their brains and decline in memory and learning abilities [19]. Thus, SerR is practically considered to synthesize d-Ser from l-Ser.

d-Asp works as a hormone-like substance and is connected to the cellular differentiation in the human body [20]. As examples of function of d-Asp in mammals, d-Asp promotes testosterone synthesis in the testicle [21] and regulates synthesis of oxytocin, vasopressin, and prolactin in the posterior pituitary gland [22,23]. In recent years, in addition to the hormone-like nature of d-Asp, its role as a neurotransmitter has been revealed [24]. d-Asp is considered to be synthesized from l-Asp via racemic reaction catalyzed by AspR. However, there is no correlation between expression levels of the *AspR*-deduced gene and concentrations of d- and l-Asp in cells, and *AspR* gene knockdown mice can still synthesize d-Asp in their cells. Therefore, the gene product of this *AspR*-deduced gene may possess no racemic activities with d- and l-Asp. Interestingly, a recent study revealed that SerR catalyzes d- and l-Asp in addition to d- and l-Ser and actually synthesizes d-Asp in mice [25]. Thus, there are still many unclear mechanisms regarding the synthesis of d-Asp in mammals.

As mentioned above, d-Ser and d-Asp are mainly synthesized by the catalysis of various enzymes in cells. However, d-amino acids derived from various foods and enteric bacteria are also taken into the human body and used [26], and therefore, the relationship between d-amino acids and d-amino-acid-producing bacteria is a highly promising research field.

## 4. d-Amino Acids in Bacteria

As mentioned above, the presence of d-amino acids in bacterial cells has been apparent for some time, but the practical roles of these d-amino acids, except for cell wall constituents, is still unclear. To date, some roles of d-amino acids, other than as a cell wall constituent, have been revealed. It was recently reported that some d-amino acids serve to prevent the formation of the biofilms of various kinds of bacteria and disassemble formed biofilms. Although inhibition of biofilm formation and disassembly of formed biofilm by d-amino acids are different mechanisms, these are often reported simultaneously [27,28,29]. The disassembly of a biofilm by d-amino acids means that d-amino acids induce a dynamic instability in a formed biofilm and lead to the breakdown of the biofilm, whereas inhibition of biofilm formation simply means preventing new biofilm formation by bacteria. As per the reports on the inhibitory effect of biofilm formation, d-leucine (d-Leu), d-methionine (d-Met), d-tyrosine (d-Tyr), d-tryptophan (d-Trp), and a mixture of these d-amino acids inhibits the biofilm formation of *Bacillus subtilis* [27] and *Staphylococcus aureus* [28], even if these d-amino acids are present at the nanomolar level (Table 1). The mixture of d-Leu, d-Met, d-Tyr, and d-Trp also exerts the inhibitory effect of biofilm formation of *Enterococcus faecalis* [30]. In addition to these d-amino acids, d-Ala and d-phenylalanine (d-Phe) inhibit some strains of *Staphylococcus epidermidis* [29]. As for the mechanism of the inhibitory effect of biofilm formation by d-amino acids, it was initially reported that these d-amino acids were incorporated into bacterial cells and used for the constituents of peptide side chains in peptidoglycan, instead of d-Ala, without inhibiting bacterial cell growth and the expression of genes related to the production of exopolysaccharide [27]. The replacement of d-Ala to d-amino acids inhibits anchoring between peptidoglycan and TasA fibers [27], which are amyloid fibers and a main constituent of biofilms. Thereafter, however, another mechanism for inhibition of biofilm formation by d-amino acids was suggested [31]. In studies on the inhibitory effect of biofilm formation by d-amino acids, *B. subtilis* strains harboring a mutated *dtd* gene encoding d-tyrosyl-tRNA deacylase, the enzyme preventing the misincorporation of d-amino acids into proteins, have been used, and the gene product of this mutated *dtd* loses its ability to prevent the misincorporation of d-amino acids into protein. When this mutated *dtd* gene is repaired to the correct sequence, the *B. subtilis* gains resistance to inhibition of biofilm formation against d-amino acids, without losing the ability of incorporation into peptidoglycan. Thus, the inhibition of biofilm formation is largely due to the misincorporation of d-amino acids into proteins. However, this suggested mechanism was investigated only in *B. subtilis*. A misincorporation of d-amino acids into peptidoglycan and other unrevealed mechanisms may be still the causes of inhibition of biofilm formation in other bacteria. The inhibition of biofilm formation by some kinds of d-amino acids varies depending on the strain because mutations in the *yqxM* gene, which is required for the formation and anchoring of the fibers to the cell, easily confer resistance to inhibition of biofilm formation by the d-amino acid [27]. A previous study tested the inhibitory effect of biofilm formation by d-Ala, d-Tyr, d-Leu, d-proline (d-Pro), d-Met, and d-Phe on *S. epidermidis* strains isolated from healthy skin, conjunctiva, and ocular infections [29]. The sensitivities of each strain to each d-amino acid were found to be very different, and biofilm formation in some strains was not inhibited by d-amino acids tested. In other examples, inhibition of biofilm formation by d-amino acids has been reported in *E*. *faecalis* [30].

On the other hand, biofilm-disassembling effects by d-amino acids have been reported in *B. subtilis* [27], *S. aureus* [28], *S. epidermidis* [29], *E. faecalis* [32], and *P. aeruginosa* [33] (Table 1). The types of d-amino acid disassembling biofilm formed by these bacteria are similar to that of d-amino acids with inhibitory effects on biofilm formation. As mentioned above, disassembly of biofilm is not same as inhibition of biofilm formation. Therefore, the mechanisms of biofilm disassembly by d-amino acids are not necessarily the same as those of the inhibition of biofilm formation. However, putative mechanisms of biofilm disassembly by d-amino acids have not yet been reported.

Despite the various studies on the inhibition of biofilm formation, several studies have found that d-amino acids have no inhibitory effect on the biofilm formation in *S. aureus* [34] and *P. aeruginosa* [35]. Bucher et al. addressed these discrepancies about inhibition of biofilm formation by d-amino acids [36] and reported that a lack of reproducibility strongly depends on various subtle experimental conditions, such as the medium composition used for the preculture, growth phase, inoculation ratio, and removal of the spent medium before the inoculation.

In any case, an inhibition of biofilm formation and disassembly of formed biofilm has been observed for some kinds of d-amino acids on various strains, and this effect has possible applications in various fields because biofilm formation is often considered a problem where sanitation is important. Indeed, despite the fact that some bacterial strains are resistant to inhibition of biofilm formation and biofilm disassembly via the misincorporation of d-amino acids, this effect has been applied in the cleaning of dental unit waterlines [37]. A five-day treatment using a mixture containing 10 mM d-Leu, d-Met, d-Tyr, and d-Trp dispersed biofilms sticking to the inside of dental unit waterlines more effectively than when 100 mM hydrochloric acid was used instead of the d-amino acid mixture. Furthermore, when a mixture of d-Met, d-Phe, and d-Trp is used along with various antibiotics, such as rifampin, colistin, and ciprofloxacin, these d-amino acids significantly enhance the antibiotic effects of these agents against the biofilm of clinical isolates *S. aureus* and *P. aeruginosa* [33].

## 5. d-Amino Acids in Fermentative Foods

As mentioned above, various kinds of d-amino acids are present in food, including raw ingredients and processed foods, such as vegetables [38], fruit [38], milk [39], and liquor [40]. Of these, fermentative foods, especially lactic acid fermentative foods, contain high amounts of d-amino acids [41], which are thought to be produced by the metabolic activities of various bacteria during the process of fermentation. The production of d-amino acids in foods by fermenting lactic acid bacteria has been experimentally demonstrated [41], and many of these d-amino acids have a sweet taste for humans, compared with the corresponding l-enantiomers [42]. Although the reason for the sweet taste has not been elucidated, the effect of d-amino acids on human gustation is important in the production of fermentative foods in terms of taste quality. Indeed, concentrations of d-Ala, d-Glu, and d-Asp in sake—a Japanese traditional rice wine—have positive correlations to its taste qualities [43], and when the same amounts of l-Ala and Dl-Ala are added to sake with a low taste quality, the addition of Dl-Ala alone does more to improve the taste [43].

In addition to improving the taste quality of foods, d-amino acids in fermentative foods are important functional components; for example, when d-Asp is ingested, d-Asp concentration in horny cell layers is increased, which serves to increase collagen and prevents cells from oxidation in the skin. Beverages containing d-Asp, for the objective of obtaining this cosmetic effect, have been practically commercialized in Japan for several years [44]. Another example of the cosmetic effect of d-Asp is black-colored vinegar made from rice and malt that contains various kinds of d-amino acids and is also commercialized in Japan. Under the current Japanese law, few d-amino acids have been approved for use as food additives as Dl-forms. Although conditions of typical vinegar production are not suitable for the growth of lactic acid bacteria, lactic acid fermentation is employed in this black-colored vinegar production.

Studies in recent years have reported various ways in which d-Asp and d-Ser may contribute to human health. For example, the oral administration of d-Asp increases its levels as identified in mice brains and enhances synaptic plasticity [45], consequently improving memory [46,47]. The d-amino acids produced by enterobacteria mitigate nephropathy in mice [48], and of the various d-amino acids made by enterobacteria, d-Ser has an especially high mitigative effect. When mice with nephropathy were dosed with d-Ser, the d-Ser dose mitigated the nephropathy of the mice, as expected. These reports are from mouse experiments and, therefore, are not necessarily applicable to humans. However, such findings may lead to the potential applications of d-amino acids as food additives or functional foods made by lactic acid fermentation. However, as mentioned above, few studies on the function of d-amino acids in humans are available, compared with l-amino acids, and many undiscovered and useful effects of various d-amino acids on cosmetic and human health are yet to be investigated. Functional foods that make use of the effects of various d-amino acids will likely appear in the future.

## 6. d-Amino-Acid-Metabolizing Enzymes in Lactic Acid Bacteria

As the d-amino-acid-producing enzymes, AlaR, AspR, and glutamate racemase (GluR), which catalyze the reversible conversion of l-amino acids to corresponding d-amino acids, are well known. Many bacteria have genes encoding these enzymes because such d-amino acids are constituents of the bacterial cell wall. Therefore, a lot of enzymatic knowledge about these racemases has been accumulated so far. However, little research about these racemases from lactic acid bacteria has been performed because the high level of d-amino acid production by lactic acid bacteria was only revealed recently. Thus, the relationship between d-amino acids and lactic acid bacteria should be further studied. Research into the enzymatic properties of these racemases has revealed a unique characteristic of AlaR from *Lactobacillus salivarius* UCC118 (LsAlaR) [49]. The presence of short-chain carboxylates, such as acetate and propionate, stabilizes and activates the LsAlaR, and these carboxylates generally inhibit the racemization activity of AlaR as substrate analogs [50]. Furthermore, A131 residue in the LsAlaR mediates the stabilization and activation effect of short-chain carboxylates in the LsAlaR. When LsAlaR racemizes l-Ala to d-Ala with 1 mM acetate in a reaction mixture, the presence of the acetate shifts the optimum reaction temperature of LsAlaR from 35 °C to 50 °C, and the A131K variant of LsAlaR loses this stabilization effect from the acetate. Another example is GluR from *Lactobacillus plantarum* NC8 (LpGluR) [51], and the large-scale production and purification method of recombinant and native-formed LpGluR has been performed using *Escherichia coli* as a host. 

As mentioned above, AlaR, AspR, and GluR are well known, and many microorganisms harbor genes encoding these racemases. However, various kinds of d-amino acids, especially d-branched chain amino acids produced extracellularly and intracellularly by some lactic acid bacteria [52], cannot be explained by the catalytic activities of these racemases with high substrate specificity. Thus, lactic acid bacteria should harbor genes encoding unique d-amino-acid-metabolizing enzymes that react to various kinds of d-amino acids, and these enzymes of lactic acid bacteria are often subjects of scholarly interest. Indeed, unique enzymes that metabolize d-amino acids have been discovered from lactic acid bacteria in recent years (Table 2).

*Lactobacillus* sp. is the most famous and representative species among lactic acid bacteria, and many of the unique enzymes that metabolize d-amino acids have been found in *Lactobacillus* sp., but only *L. salivarius*, which produces especially high amounts of d-Ala in cells [53], harbors the deduced d-amino acid amino transferase (DAAT) gene, and the DAAT activity of its gene products has been demonstrated in previous research [53]. The DAAT of *L. salivarious* (LsDAAT) has unique characteristics, compared with the other DAATs previously characterized. Despite the fact that the DAAT of *Bacillus* sp. YM-1 [58], *B. subtilis* NRRLB 1471 [59], *Lysinibacillus sphaericus* [60], and *Geobacillus toebii* SK1 [61] show little or no substrate specificity to d-branched chain amino acids, such as d-*allo*-isoleucine (d-*allo*-Ile), d-Leu, and d-valine (d-Val), LsDAAT shows high activities to these d-branched chain amino acids as amino donors (Table 3). Furthermore, the LsDAAT has a very broad spectrum of amino acceptor specificities. Although these unique characteristics of LsDAAT have been established, the relationship between the LsDAAT and practical d-amino acid production by *L. salivarius* is still unclear. As mentioned above, the DAAT gene exists only in *L. salivarius* within *Lactobacillus* sp., so the unique d-amino acid production by *Lactobacillus* sp. cannot be explained by the unique characteristics of DAAT.

With regard to d-branched chain amino acids, Mutaguchi et al. found that some lactic acid bacteria produce various kinds of d-branched chain amino acids and secrete them in the growth medium [54], and these lactic acid bacteria have no deduced DAAT gene. They also found that crude extracts of these lactic acid bacteria engage in racemization activities for various kinds of d-branched chain amino acids [54] and successfully purified this deduced racemase, catalyzing d-branched chain amino acids from a crude extract of *Lactobacillus otakiensis* JCM 15040. Through the N-terminal amino acid analysis of the purified enzyme, the encoding gene seems to be annotated as γ-aminobutyrate aminotransferase in the *Lactobacillus buchneri* JCM 1115 genome. Mutaguchi et al. prepared the recombinant protein deduced as the γ-aminobutyrate aminotransferase of *L. buchneri* JCM 1115 using the pET system in *E*. *coli* cells, and the purified recombinant enzyme isoleucine 2-epimerase (ILEP) showed a racemization activity among various kinds of d- and l-amino acids, especially branched chain amino acids (Table 2 and Table 4). Thus far, amino acid racemase with a broad spectrum of substrate specificity has been reported only in *Pseudomonas* sp. [62] and hyperthermophile *Pyrococcus horikoshii* OT-3 [63], except for *Lactobacillus* sp., and these racemases have low or almost no specific activities for d-branched chain amino acids (Table 4). Furthermore, lactic acid bacteria carrying these ILEP homolog genes extracellularly produce d-branched chain amino acids in the exponential, early stationary, and stationary phases [52]. These results strongly imply that the ILEP is practically involved in the production of d-branched chain amino acids in these lactic acid bacteria.

Another example of a unique enzyme that uses d-amino acids as substrates was recently discovered in lactic acid bacteria: lysine racemase from the *Oenococcus oeni* PSU-1 strain (OoLysR) (Table 2) [55]. The OoLysR exhibited a racemic activity for l-arginine (l-Arg) and l-ornithine (l-Orn), in addition to l-lysine (l-Lys). Interestingly, although the OoLysR showed approximately 60% sequence similarity to AlaR of *G. stearothermophilus* (GsAlaR), the OoLysR and GsAlaR cannot racemize d- and l-Ala and d- and l-Lys, respectively, and *OoLysR* gene was annotated as AlaR at first. The authors elucidated a part of this difference of substrate specificity between OoLysR and GsAlaR by preparing an OoLysR variant protein with T224I and/or W355Y replacements. These two residues are located on the active site and conserved as I222 and Y354 in GsAlaR. The prepared OoLysR^T224I^, OoLysR^W355Y^, and OoLysR^T224I/W355Y^ variant enzymes showed very weak racemization activities toward d- and l-Ala, and the GsAlaR variant with I222T and/or Y354W replacements showed weak but apparent racemization activities toward d- and l-Lys. Although the substrate specificity of AlaR is generally strict, these results suggest that the residues in the active site of AlaR play a very important role for substrate recognition, and therefore research indicating that only one residue replacement confers the racemic activity of d- and l-Lys on AlaR may yield insight into the evolution of various d-amino-acid-producing microorganisms.

The unique enzyme that metabolizes various kinds of d-amino acids was identified in *Lactobacillus sakei* LT-13 (Table 2 and Table 4) [56]. The gene that encodes this enzyme was initially annotated as the cystathionine β-lyase homolog gene (*malY*). However, its gene product (MalY) exhibits racemic activities to various kinds of d-amino acids and β-lyase activity to l-cysteine (l-Cys) simultaneously (Table 2 and Table 4). The racemic activities of MalY from *L. sakei* LT-13 (LsMalY) are apparently different to other, already-known racemases, including ILEP from *L. buchneri* JCM 1115 (Table 4). The substrate specificity of LsMalY is relatively similar to AlaR from *Pseudomonas putida* IFO 12996 in terms of the activities to l-Arg and l-histidine (l-His). Interestingly, *L. sakei* LT-13, which has the *LsMalY* gene, is a low d-amino acid producer, and the high d-amino acid producer *L. sakei* LT-145 does not possess the *LsMalY* corresponding gene. Therefore, the practical d-amino acid production by LsMalY and the intracellular function of LsMalY are still unclear.

In the *E. coli* genome, the *MalY* gene (*EcMalY*) is regarded as the regulator of the maltose repressor, which interacts with the MalT protein [64]. However, the ligands for EcMalY are unidentified, and therefore, the finding that LsMalY catalyzes d-amino acids may lead to new discoveries with regard to how d-amino acids function in vivo.

Another recent finding is that histidine racemase (HisR) is present in *Leuconostoc mesenteroides* subsp. sake NBRC 102480, which is a lactic acid bacterium isolated from Kimoto in sake, and reported for the first time as d- and l-His specific racemase (Table 2) [57]. The recombinant protein of HisR from *L. mesenteroides* subsp. sake NBRC 102480 (LmHisR) has shown a highly specific racemization activity to d- and l-His. Because d-His is known to be rare in nature, the authors deduced that the physiological function of the LmHisR is not to catabolize d-His but rather to produce d-His and that d-His is an essential component of *L. mesenteroides* subsp. sake NBRC 102480. However, the practical roles of d-His in *L. mesenteroides* subsp. sake NBRC 102480 are still unknown.

In this way, unique d-amino-acid-metabolizing enzymes have been identified in various lactic acid bacteria in recent years, and some of them are likely to be involved in practical d-amino acid production. However, the reason why some lactic acid bacteria extracellularly produce these various d-amino acids and the practical functions of these enzymes in d-amino acid synthesis are still mostly unclear. To address these issues, the disruption of the genes encoding these enzymes should be considered. However, the gene disruption method for lactic acid bacteria is still underexplored, and future studies will be beneficial in this regard. As mentioned above, researchers have proposed a number of useful effects of d-amino acids for cosmetic and human health, and they are likely to be commoditized, giving rise to the possibility that the industrial-scale production of d-amino acids will take place in the future. It this comes to pass, d-amino-acid-metabolizing enzymes will be needed for the mass production of d-amino acids. Therefore, unique d-amino-acid-metabolizing enzymes should be explored not only to elucidate the function of d-amino acid in organisms but also to enable the mass production of d-amino acids.

## Figures and Tables

**Table 1:** Reported bacteria whose biofilm is disassembled by d-amino acids.

Bacterium	d-Amino Acid Disassembling Biofilm	d-Amino Acid Inhibiting Biofilm Formation
*B. subtilis* [27]	d-Tyr, d-AA mixture (d-Leu, d-Met, d-Trp, and d-Tyr)	d-Leu, d-Met, d-Trp, and d-Tyr
*E. faecalis* [30]	Not described	d-AA mixture (d-Leu, d-Met, d-Trp, and d-Tyr)
*E. faecalis* [32]	d-Leu	Not described
*P. aeruginosa* [32]	Not described	d-Tyr and d-AA mixture (d-Leu, d-Met, d-Trp, and d-Tyr)
*P. aeruginosa* [33]	d-Met, d-Phe, and d-Trp	Not described
*S. aureus* [27]	Not described	d-Tyr and d-AA mixture (d-Leu, d-Met, d-Trp, and d-Tyr)
*S. aureus* [28]	d-AA mixture (d-Phe, d-Pro, and d-Tyr)	d-Leu, d-Met, d-Phe, d-Pro, d-Trp, and d-Tyr
*S. aureus* [33]	d-Met, d-Phe, and d-Trp	Not described
*S. epidermidis* [29]	d-Met, d-Phe, and d-Pro	d-Leu, d-Tyr, d-Pro, d-Phe, d-Met, and d-Ala

d-AA represents d-amino acid. The effects of biofilm disassembly and inhibiting biofilm formation of each d-amino acid differ in various factors, such as d-amino acid concentrations, strains, and growth conditions.

**Table 2:** Recently reported d-amino-acid-metabolizing enzymes of lactic acid bacteria.

Bacteria	*L. sal* [49]	*L. pla* [51]	*L. sal* [53]	*L. buc* [54]	*O. oen* [55]	*L. sak* [56]	*L. mes* [57]
Enzyme	Alanine racemase	Glutamate racemase	d-Amino acid transaminase	Amino acid racemase (Isoleucine 2-epimerase)	Lysine racemase	Bifunctional amino acid racemase	Histidine racemase
Optimum temperature (°C)	35 (l-Ala) 50 (l-Ala)	50 (d-Glu)	60 (d-Ala and 2-OG)	50 (l-Ile) 45 (d-*allo*-Ile)	-	45 (l-Ala) as racemase 40 (l-Cys) as β-lyase	50 (l-His)
Optimum pH	8.5 (l-Ala) 8.5 (l-Ala)	8.0 (d-Glu)	6.0 (d-Ala and 2-OG)	5.0 (l-Ile) 6.0 (d-*allo*-Ile)	9.0 (l-Lys)	10 (l-Ala) as racemase 10 (l-Cys) as β-lyase	9.5 (l-His)
*K_m_* (mM)	11.5 (l-Ala)	1.64 (d-Glu) 0.84 (l-Glu)	1.05 (d-Ala) 3.78 (2-OG)	5.00 ± 0.080 (l-Ile) 13.2 ± 0.644 (d-*allo*-Ile)	11 ± 1 (l-Lys) 9.8 ± 1.5 (d-Lys) 27 ± 4 (l-Orn) 22 ± 2 (d-Orn)	169 ± 27 (l-Ala) as racemase 150 ± 24 (d-Ala) as racemase 11.5 ± 1.1 (l-Cys) as β-lyase	8.14 (l-His) 17.26 (d-His)
*V_max_*	272 μmol min^–^^1^ mg^–^^1^	-	-	-			-
*K_cat_* (s^–^^1^)	-	5980 (d-Glu) 3029 (l-Glu)	-	502 ± 16.2 (l-Ile) 939 ± 26.8 (d-*allo*-Ile)	3.5 ± 0.2 (l-Lys) 2.8 ± 0.2 (d-Lys) 1.8 ± 0.5 (l-Orn) 1.8 ± 0.2 (d-Orn)	(2.31 ± 0.51) × 10^3^ (l-Ala) as racemase (1.83 ± 0.15) × 10^3^ (d-Ala) as racemase (9.30 ± 2.05) × 10^2^ (l-Cys) as β-lyase	96.95 (l-His) 185.42 (d-His)
Temperature stability (°C)	-	20 ^a^	45 ^b^	60 ^b^	60 ^c^	-	-
pH stability	-	8.0 ^a^	5.5–10.0 ^b^	5.0–11.0 ^b^	-	-	-
Substrate specificity	d- and l-Ala specific	d- and l-Glu specific	Broad	Broad	Reacts to l-Arg and l-Orn	-	d- and l-His specific
Molecular mass (kDa)	43	29.7 (Homodimer)	31.5 (Homodimer)	49 (Homotetramer)	-	-	42 (Homodimer)
Cofactor	PLP	none	PLP	PLP	PLP	-	-
Notable characteristic	Stabilized in the presence of carboxylates, such as acetate and propionate, and this feature is caused by the A131 residue	-	Broad amino donor and acceptor specificity, especially d-branched amino acids	Broad substrate specificity, especially d-branched amino acids	-	Broad substrate specificity, β-lyase activity simultaneously exists	The first identification and characterization

–: The data are unavailable. The substrates are represented in parenthesis. ^a^ More than 80% residual activity for 20 minutes’ incubation. ^b^ More than 80% residual activity for 30 minutes’ incubation. ^c^ More than 80% residual activity for 60 minutes’ incubation. 2-OG and PLP represent 2-oxoglutarare and pyridoxal 5-phosphate, respectively. *L. sal*: *Lactobacillus salivarius* UCC118; *L. pla*: *Lactobacillus plantarum* NC8; *L. buc*: *Lactobacillus buchneri* JCM 1115; *O. oen*: *Oenococcus oeni* PSU-1; *L. sak*; *Lactobacillus sakei* LT-13; *L. mes*: *Leuconostoc mesenteroides* subsp. sake NBRC 102480.

**Table 3:** Substrate specificity of various d-amino acid aminotransferase.

d-Form substrate	*L. sal* [53]	*Bacillus* sp. [58]	*B. sub* [59]	*L. sph* [60]	*G. toe* [61]
Ala	100 ^a^	100 ^b^	100 ^c^	100 ^b^	100 ^b^
*allo*-Ile	104	-	-	<1	-
Abu	90	98	100	97	89
Met	89	19	14	61	1
Leu	84	2	<1	8	-
Nva	83	34	50	83	8
Val	83	3	<1	<1	2
His	75	2	-	6	2
Nle	71	5	-	46	-
Pgl	41	-	-	-	-
Thr	13	<1	<1	<1	1
Arg	5	1	-	6	-
Ser	<1	60	7	30	70
Cys	<1	25	<1	31	11
Pro	<1	1	-	<1	12
Asn	<1	1	62	32	-
Ile	<1	<1	<1	<1	-
*allo*-Thr	<1	<1	-	<1	-
Phe	<1	<1	<1	<1	-
β-Ala	<1	<1	-	<1	-
Asp	<1	-	41	-	-
Trp	-	-	<1	-	-
Lys	-	-	<1	-	-

–: The data are unavailable. 2-Oxoglutarate was used as the amino acceptor. ^a^ 24.1 ± 1.31 μmol min^–1^ mg^–1^. ^b^ The specific activity at 100% relative activity was not written. ^c^63 μmol min^–1^ mg^–1^. Abu, Nva, Nle, Pgl, Thr, and Asn represent α-amino butyrate, norvaline, norleucine, pyroglutamate, threonine, and asparagine. *L. sal*: *Lactobacillus salivarius* UCC118; *Bacillus* sp.: *Bacillus* sp. YM-1; *B. sub*: *Bacillus subtilis* NRRLB 1471; *L. sph*: *Lysinibacillus sphaericus* IFO 3525; *G. toe*: *Geobacillus toebii* SK1.

**Table 4:** Substrate specificity of various amino acid racemases with broad substrate specificity.

l-Form substrate	*L. buc* [54]	*P. put* [62]	*P. hor* [63]	*L. sak* [56]
Ile	100 ^a^	<1	48	<1
Nva	56	-	-	-
Nle	50	-	-	-
Val	48	<1	13	27
Abu	32	-	-	-
Leu	30	3	86	1
Phe	24	<1	100 ^c^	<1
Met	21	14	64	20
*allo*-Ile	19	-	-	<1
Ser	6	20	10	19
Ala	3	33	5	100 ^b^
Arg	<1	65	<1	82
Asn	<1	<1	<1	8
Asp	<1	<1	<1	<1
Gln	<1	-	<1	6
Glu	<1	-	<1	<1
His	<1	2	<1	15
Lys	<1	100 ^b^	-	10
Orn	<1	-	-	-
*tert*-Leu	<1	-	-	-
Thr	<1	<1	42	<1
Trp	<1	<1	27	2
*allo*-Thr	-	-	-	<1
Cys	-	14	-	-
Pro	-	<1	-	<1
Tyr	-	-	-	<1

–: The data are unavailable. ^a^ 149 ± 4.01 μmol min^–1^ mg^–1^. ^b^ 132 μmol min^–1^ mg^–1^. ^c^ The specific activity at 100% relative activity was not written. ^d^ 0.0093 ± 0.0003 μmol min^–1^ mg^–1^. Gln represents glutamine. *L. buc*: *Lactobacillus buchneri* JCM 1115; *P. put*: *Pseudomonas putida* IFO 12996; *P. hor*: *Pyrococcus horikoshii* OT-3; *L. sak*: *Lactobacillus sakei* LT-13.
